# Role of the Melanocortin System in the Central Regulation of Cardiovascular Functions

**DOI:** 10.3389/fphys.2021.725709

**Published:** 2021-08-25

**Authors:** Francesca Copperi, Jung Dae Kim, Sabrina Diano

**Affiliations:** ^1^Institute of Human Nutrition, Columbia University Irving Medical Center, New York, NY, United States; ^2^Department of Molecular Pharmacology and Therapeutics, Columbia University Irving Medical Center, New York, NY, United States; ^3^Department of Physiology and Cellular Biophysics, Columbia University Irving Medical Center, New York, NY, United States

**Keywords:** melanocortin system, cardiovascular functions, α-MSH, γ-MSH, MC3R, MC4R

## Abstract

Increasing evidence indicates that the melanocortin system is not only a central player in energy homeostasis, food intake and glucose level regulation, but also in the modulation of cardiovascular functions, such as blood pressure and heart rate. The melanocortins, and in particular α- and γ-MSH, have been shown to exert their cardiovascular activity both at the central nervous system level and in the periphery (e.g., in the adrenal gland), binding their receptors MC3R and MC4R and influencing the activity of the sympathetic nervous system. In addition, some studies have shown that the activation of MC3R and MC4R by their endogenous ligands is able to improve the outcome of cardiovascular diseases, such as myocardial and cerebral ischemia. In this brief review, we will discuss the current knowledge of how the melanocortin system influences essential cardiovascular functions, such as blood pressure and heart rate, and its protective role in ischemic events, with a particular focus on the central regulation of such mechanisms.

## The Melanocortin System and Cardiovascular Functions

The melanocortin system consists of several melanocortin peptides and their receptors expressed in the brain, as well as in the periphery. In the central nervous system (CNS), the proopiomelanocortin (POMC) neurons produce several peptides by post-translational modification, such as adrenocorticotrophin (ACTH), α-,β-, and γ-melanocyte stimulating hormone (α-MSH, β-MSH, and γ-MSH), and β-endorphin (Smith and Funder, 1988). These melanocortin peptides bind to five melanocortin receptor subtypes (MC1R-MC5R) with differential binding affinity (Kim et al., 2002). Among melanocortin peptides, α-MSH is the most well-known anorexigenic peptide, able to inhibit food intake and increase energy expenditure mainly through central MC4R activation (Ollmann et al., 1997). Unlike α-MSH, γ-MSH has stronger affinity for MC3R than MC4R. Among the five MCRs, MC3R, and MC4R are predominantly expressed in several brain nuclei and play a central role in the regulation of food intake and energy expenditure (Seeley et al., 1997; Cone, 2005; Sohn et al., 2013a). The activity of POMC neurons is opposed by Neuropeptide Y/Agouti-related peptide (NPY/AgRP) expressing neurons, which produce the MC3R and MC4R inverse agonist AgRP (Ollmann et al., 1997).

In the brain, POMC expressing neurons are predominantly located in the arcuate nucleus (ARC) of the hypothalamus and the nucleus tractus solitarius (NTS) of the brainstem (Young et al., 1998). Neighboring the ARC POMC neurons, NPY/AgRP expressing neurons are located on the more ventromedial area of the ARC.

Both ARC and NTS POMC neurons regulate feeding and energy homeostasis by sensing long-term adiposity signals and short-term satiety signals respectively (Zhan et al., 2013). Consistently, mutations of the POMC gene cause hyperphagia leading to obesity in both mice and humans (Yaswen et al., 1999; Challis et al., 2004).

In addition to their well-known effects on food intake and energy homeostasis, the melanocortin system has been reported to be able to influence multiple cardiovascular functions. In particular, melanocortin peptides can regulate blood pressure (BP) and hearth rate (HR) both as circulating hormones via numerous neurohumoral and renal mechanisms, and centrally by acting within the CNS and modulating sympathetic nerve activity (SNA). Finally, melanocortin peptides have been implicated in influencing the outcome of certain important cardiovascular diseases, such as myocardial and cerebral ischemia.

In this review we will focus on revising the current knowledge about the role of melanocortin peptides and receptors in cardiovascular function, with particular attention to the central regulatory mechanisms.

## Effects of the Melanocortin System on Blood Pressure and Heart Rate

**FIGURE 1:**
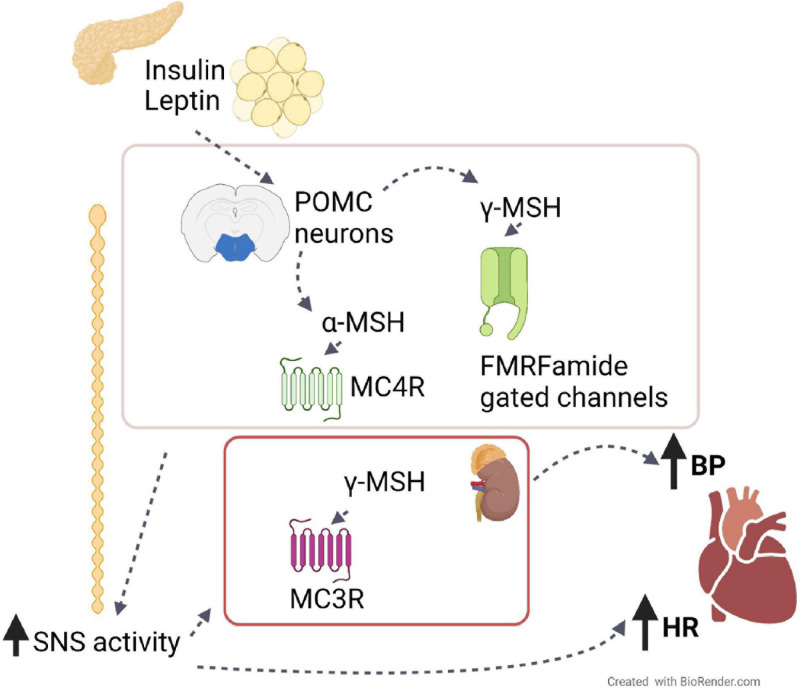
The role of the melanocortin system on BP and HR. Leptin from adipose tissue and insulin from the pancreas induce the production from hypothalamic POMC neurons of melanocortin peptides, such as α- and γ-MSH. By binding their receptors, MC4R and FMRFamide gated ion channels, respectively, these peptides induce the activation of the sympathetic nervous system, which in turn increases blood pressure (BP) and heart rate (HR). The same effects are also caused peripherally by γ-MSH via its binding to receptor MC3R in the kidney.

Although systemic α-MSH administration does not affect BP (Kuo et al., 2004; Ni et al., 2006), numerous studies have shown that acute intracerebroventricular (ICV) injection of α-MSH can increase BP via an increase in the SNA (Hill and Dunbar, 2002; Matsumura et al., 2002) (Figure 1). In conscious rats, acute ICV administration of α-MSH has been shown to significantly increase the mean arterial pressure (MAP) and HR for 1 h, returning to control levels after 2 h from its administration. On the other hand, chronic ICV infusion of α-MSH was shown to increase MAP during the dark period (active phase) for the first 2 days, while significantly decreasing for the following 5 days compared to controls. On the other hand, the HR was significantly higher compared to controls throughout the whole monitoring period (9 days), accompanied by reduced physical activity and food intake (Hill and Dunbar, 2002). The central effects of α-MSH on BP were shown to be dependent on MC4R, as they were completely abolished in *Mc4r* knock out animals (Ni et al., 2006). Similar to what was observed by ICV administration of α-MSH, chronic ICV infusion of the MC3/4R agonist MTII significantly increased MAP during the 14-day experimental period. Consistently, chronic infusion of the MC3/4R antagonist SHU-9119 significantly reduced HR and showed a tendency to decrease MAP (Kuo et al., 2003). Interestingly, in anaesthetized rats, direct injection of MTII into the paraventricular nucleus of the hypothalamus (PVN) increased renal SNA and MAP, and its effects were abolished by AgRP or SHU9119 (both being MC3/4R antagonists) pretreatment into the PVN (Li et al., 2013). However, in such studies performed on anaesthetized rats, the magnitude of MTII effects on MAP was smaller than that observed in conscious rats (∼4 mmHg vs. ∼10 mmHg increase), suggesting that a fully functional SNS might be required for maximum effects of MCR activation on MAP. Indeed, the increase in BP observed upon chronic activation of MC4R is completely abolished by α/β-adrenergic receptors blockade, indicating that such effect is mediated by SNS activity (Kuo et al., 2004).

The effects of melanocortin receptor activation and inhibition has been largely investigated in obesity-induced hypertension, a condition of great clinical relevance since about 85% of individuals with metabolic syndrome have hypertension (Franklin, 2006). In obese Zucker rats, antagonism of MC4R caused significantly greater reduction in BP compared to lean control animals (do Carmo et al., 2012). In humans, *Mc4r* loss-of-function mutations account for about 5% of all early-onset obesity cases (Yeo et al., 1998; Vaisse et al., 2000). Obese patients with *Mc4r* mutations have smaller prevalence of hypertension, reduced BP and reduced norepinephrine secretion, despite being severely obese and showing severe metabolic abnormalities, such as hyperphagia and hyperinsulinemia (Greenfield et al., 2009; Greenfield, 2011). In these patients presenting *Mc4r* haploinsufficiency, administration of the melanocortin agonist LY2112688 induced significant increase in BP without changes in insulin levels (Greenfield et al., 2009).

Similar results were observed in lean forms of hypertension. In spontaneously hypertensive rats (SHR), in which the increased BP is caused by hyperactivation of SNS, chronic antagonism of central MC4R caused greater BP reduction than in lean normotensive rats, in a similar way to what was observed upon α/β-adrenergic receptors blockade, despite causing hyperphagia and weight gain (Da Silva et al., 2008). Finally, the MC3/4R antagonist SHU-9119 was able to significantly decrease BP in pharmacological-induced hypertension upon chronic infusion of nitric oxide synthase inhibitor (L-NAME) (Da Silva et al., 2015). However, the same study also showed that MC4R antagonism was not effective in lowering BP in angiotensin-II-induced hypertension, indicating MC3/4R-independent mechanisms in SNA modulation (Da Silva et al., 2015).

Different to what was observed with α-MSH and MTII treatments, injection of γ-MSH originated inconsistent results. Intravenous (IV) or ICV γ-MSH injections raised BP and HR, whereas its injection specifically in the NTS had opposite effect (Li et al., 1996; Humphreys et al., 2011). The effects of γ-MSH in elevating BP were independent of MC3/4R, as demonstrated by the inability of AgRP or synthetic antagonists, as well as the lack of MC3R and MC4R to counteract γ-MSH treatment effects (Li et al., 1996). On the other hand, ICV administration of benzamil, an amiloride analog, completely blunted γ-MSH effect, suggesting that the γ-MSH-dependent increase in BP is mediated by Phe-Met-Arg-Phe-NH2 (FMRFamide) gated ion channels rather than by MCRs (Ni et al., 2006) (Figure 1). Additionally, it has also been shown that γ-MSH can exert its effects on regulating BP peripherally. Indeed, IV γ-MSH infusion lowered MAP in high sodium diet-fed (HSD) hypertensive animal models (Mayan et al., 2003; Ni et al., 2003). The ability of γ-MSH in lowering MAP was more effective in HSD-fed than Dahl salt-resistant (DSR) rats and was mediated by the natriuretic effect of γ-MSH via renal MC3R activation rather than by central mechanisms (Chandramohan et al., 2009) (Figure 1).

In addition to the prominent role of MC4R and MC3R in regulation of BP and HR from the CNS, other melanocortin receptors have been shown to affect cardiovascular functions. In particular MC1R and MC2R can regulate arterial stiffness (Rinne et al., 2015), atherosclerosis (Rinne et al., 2018) and vascular smooth muscle cell proliferation (Tang et al., 2018). However, their effects seem to be autocrine or paracrine rather than mediated by the CNS.

Even though the role of ACTH in regulating BP has been known for over 30 years (Whitworth et al., 1983), the exact mechanisms through which this melanocortin exerts its effect are not yet fully understood. Early studies observed that administration of ACTH was able to increase BP in both normotensive and hypertensive human subjects (Whitworth et al., 1983). Additionally, pathological conditions characterized by high levels of circulating ACTH, such as Cushing’s diseases, have been associated with hypertension (Cicala and Mantero, 2010). Numerous evidence suggest that the ability of ACTH in raising BP is mediated by the adrenal gland and its secretion of glucocorticoids and mineralocorticoids hormones, and involves natriuresis regulation by the kidneys and regulation of vascular tone (Connell et al., 1987; Woods et al., 1988; Hatakeyama et al., 2000). However, ACTH was also shown to be able to increase BP independently of increase in glucocorticoids, enhancing the effects of norepinephrine and angiotensin II (Woods et al., 1988).

Finally, early observations pointed at the correlation of increased plasma levels of another melanocortin, β-endorphin, and hypertension (Guasti et al., 1996). Following studies showed that administration of β-endorphin decreased BP and affected the hormonal profile of both healthy normotensive and hypertensive individuals, via opioid receptors (Cozzolino et al., 2005), similarly to what previously observed in rats (Sitsen et al., 1982).

## Brain Nuclei Involved in Melanocortin-Mediated Cardiovascular Regulation

Both MC3R and MC4R are broadly expressed in the CNS (Mountjoy, 2010). Even though most of the studies used IV or ICV administration of pharmacological melanocortin receptor agonists and antagonists, few studies unraveled the specific brain regions involved in regulating BP and HR via melanocortin receptors. Among the others, the paraventricular nucleus of the hypothalamus (PVN) is of central importance in regulating numerous MC4R-dependent metabolic functions such as food intake and thermogenesis (Fan et al., 1997; Huszar et al., 1997). Additionally, direct administration of the MC4R agonist MTII into the PVN was able to significantly increase MAP and HR by modulating the renal SNS outflow (Li et al., 2013). Similar effect of MC4R activation could also be observed in hyperinsulinemia-induced hypertension (Ward et al., 2011). Within the hypothalamus, the dorsomedial hypothalamus (DMH) was also shown to be involved in the increase in BP caused by higher leptin levels in diet-induced obesity, possibly via MC4R (Simonds et al., 2014). Tachycardia was also induced by α-MSH injection in the intermediolateral medulla (Iwasa et al., 2013), as well as by MTII administration in the parabrachial nucleus and rostral ventrolateral medulla, indicating that such functions are not limited to hypothalamic nuclei (Skibicka and Grill, 2009). Interestingly, α-MSH and MTII injection in the NTS caused bradycardia, opposite to what was observed for other nuclei, indicating that the effects on cardiovascular functions of MC4R activation in the CNS is not uniform (Tai et al., 2007). Finally, within the brain stem, cholinergic neurons of the DMV are inhibited by MC4R activation leading to decreased parasympathetic activity, whereas, in the spinal cord, IML cholinergic neurons are activated via MC4R inducing increased sympathetic activity. Thus, re-expression of MC4R in cholinergic neurons of MC4R knockout mice led to obesity-induced hypertension (Sohn et al., 2013b).

## The Melanocortin System for Cardiovascular Protection

In addition to their role in regulating HR and BP, numerous studies have shown that melanocortins, mostly via MC3R and MC4R, exert important cardiovascular protective functions. Their protective role has been particularly well characterized in animal models of cardiac and cerebral ischemia, respectively, the first and second main causes of death globally (World Health Organization, 2019). In particular, following myocardial ischemia and the consequent cardiac reperfusion, which are events characterized by high lethality due to the induction of arrhythmia (Manning and Hearse, 1984), IV administration of α-MSH and γ-MSH were able to significantly prevent ventricular tachycardia and ventricular fibrillation (Bazzani et al., 2001; Guarini et al., 2002). γ-MSH was also able to completely prevent the MAP fall normally observed following coronary reperfusion. Such effects were still observed when MC4R was antagonized, but not with concomitant treatment of MC3R antagonist, indicating that they are mediated by MC3R rather than MC4R (Guarini et al., 2002). Interestingly, whereas early studies suggested that the protective effects of melanocortins might be due to inhibition of overproduction of reactive oxygen species (Bazzani et al., 2001) and reduction of inflammation (Wikberg, 1999) at the ischemic site, following data have indicated that this improved outcome was, at least partially, mediated by the CNS (Bazzani et al., 2002). Indeed, ICV administration of ACTH upon induced myocardial ischemia and reperfusion in rats was able to reduce the incidence of ventricular tachycardia, ventricular fibrillation and lethality, and prevent the fall in MAP at a dose 10 times lower than when administrated IV (Bazzani et al., 2002). Similarly, ICV infusion of the MC4R agonist MTII in rats following myocardial ischemia improved cardiac structure and function, and attenuated the fall in HR. The same effects were observed following ICV leptin infusion, but not in absence of MC4R, indicating that these effects are mediated by the leptin-melanocortin axis (Gava et al., 2021).

Cerebral ischemia is a life-threatening event in which the occlusion of a blood vessel by a blood clot leads to irreversible neurological damage. Currently the main way to treat this consists of the administration of thrombolytic agents within up to 3 h from the occurrence, posing risk of cerebral hemorrhage and with limited benefit (Powers et al., 2019). Remarkably, α-MSH analog systemic chronic treatment was able to improve the outcome of transient cerebral ischemia in gerbils and rats by reducing tissue damage and neuronal loss, and by improving functional recovery. This was seen particularly within the hippocampus, a region involved in spatial learning and memory, that is also particularly sensitive to ischemia-induced cell death (Giuliani et al., 2006, 2007). Such beneficial effects were prevented by pre-treatment with MC4R antagonist, indicating that they are MC4R-mediated (Giuliani et al., 2006, 2007). Interestingly, in addition to the antiapoptotic effects of MC4R activation, treatment with α-MSH analog enhanced learning in ischemic gerbils compared to non-ischemic controls (Giuliani et al., 2006). This was possibly due to an increase in neurite sprouting and functional recovery from nerve injury, which improves neuronal plasticity and reliance on the undamaged hemisphere, which are typical processes of stoke recovery (Pekna et al., 2012).

Treatment with high dose of ACTH has also been reported to have life-saving effects in improving cardiovascular function following aortic dissection (Noera et al., 2001), an uncommon but mostly fatal condition in which a tear in the inner layer of the aorta causes its separation from the outer layer, often causing its rupture. Following studies in rats showed that this seems to be not mediated by the adrenal gland, but rather mediated by the descending vagal pathway following central MC4R activation (Guarini et al., 2004).

The remarkable effects of the melanocortin system in regulating cardiovascular function, however, have been long standing obstacle to the use of melanocortin system-modulating compounds for the treatment of other conditions such as obesity, without affecting HR and BP. Recently, the MC4R agonist Setmelanotide has been approved by the Food and Drug Administration for the treatment of obesity. Interestingly, patients receiving Setmelanotide did not show any change in cardiovascular function, HR and BP (Chen et al., 2015; Kühnen et al., 2016; Clément et al., 2018).

## MC4R-Mediated Leptin and Insulin Effects on Cardiovascular System

Leptin is an anorexigenic hormone produced by adipocytes (Zhang et al., 1994; Frederich et al., 1995). Circulating leptin conveys information of the body energy status to the brain, enabling it to maintain the normal energy balance by acting on leptin receptors (ObRa-ObRf). Deficiency of leptin and leptin receptors leads to a morbid obese phenotype in both rodents and humans (Halaas et al., 1995; Chen et al., 1996; Montague et al., 1997; Clément et al., 1998). Interestingly, obese individuals with leptin gene mutations and thus, lower leptin levels, do not show the typical hypertension shown by individuals with the metabolic syndrome, but rather show hypotension (Mark et al., 1999; Ozata et al., 1999). In support of leptin’s role in regulating BP, chronic leptin infusion in lean rats induces an increase in BP (Carlyle et al., 2002) and an increase SNS activity (Kalil and Haynes, 2012). However, in diet-induced obesity characterized by leptin resistance, mice do display hypertension, which was shown to be blunted by antagonism of ObR in a selective area of the hypothalamus, the dorsomedial nucleus, thus suggesting that this area is spared from leptin resistance (Simonds et al., 2014). In addition, through its receptors, leptin also requires a functional central melanocortin system, including POMC neurons and MC4R, for its effects on renal SNA and BP regulation (Tallam et al., 2005, 2006; da Silva et al., 2014; Samuelsson et al., 2016). Deletion of ObRs in POMC neurons abolished both the anorexigenic and the BP lowering effects of leptin (Do Carmo et al., 2011), and leptin-driven enhancement in renal SNA was abolished in MC4R deficient mice (Rahmouni et al., 2003). Consistently, leptin-induced increase in MAP and HR was completely blocked by chronic ICV infusion of MC3/4R antagonist SHU-9119 in rats (Da Silva et al., 2004). Thus, leptin’s ability to increase BP via SNS activity is mediated by increased POMC neuronal activity and MC4R activation (Da Silva et al., 2013) (Figure 1). Finally, in addition to the hypothalamic arcuate and the dorsomedial nuclei, in obese rabbits, leptin has been shown to regulate the cardiovascular function by acting directly on the ventromedial nucleus of the hypothalamus (VMH), as intra-VMH injections of a leptin receptor antagonist or a MC3/4R antagonist, decreases MAP, heart rate, and RSNA compared to vehicle injected HFD rabbits (Lim et al., 2016).

Insulin, upon release from the pancreas, does not only regulate glucose homeostasis by promoting glucose absorption, but also exerts important effects on energy balance and food intake through the insulin receptors in the ARC (Bruning et al., 2000; Obici et al., 2002). Such CNS effects of insulin on food intake are mediated by the melanocortin system, as indicated by their reduction upon ICV administration of MC4R antagonist (Benoit et al., 2002). Additionally, insulin is known to have significant effects on BP regulation by increasing renal sodium reabsorption, inducing renal SNA, altering transmembrane ion transport, and inducing hypertrophy of blood vessels (Salvetti et al., 1993). Consistently, similar to what was observed with leptin, ICV administration of insulin raises BP by increasing SNA to the kidney (Muntzel et al., 1995; Huang et al., 1998). There is much evidence to indicate that insulin’s effect in increasing BP is mediated by the melanocortin system. Indeed, obese patients with *Mc4r* mutations present attenuated insulin-mediated SNA (Greenfield, 2011), and the increase in renal SNA observed upon ICV insulin administration in mice was completely abolished in *Mc4r* knockout mice, indicating that this effect is mediated by MC4R (Rahmouni et al., 2003).

## Conclusion

The melanocortin system is a powerful regulator of cardiovascular function within the CNS. Additionally, by being one of the main central players in modulating metabolic functions such as food intake and energy expenditure, and the subsequent development of obesity, it offers an important link between metabolic and cardiovascular diseases, two of the leading causes of mortality, morbidity and long term disability worldwide (James et al., 2018). Multiple studies reviewed here have highlighted how the central melanocortin system, and in particular the antagonism of the MC3R and MC4R receptors, are able to significantly decrease elevated BP and HR in both lean and obese forms of hypertension. Consistently, clinical observation of obese *Mc4r* haploinsufficient patients showed that MC4R loss-of-function causes a significantly lower prevalence of hypertension. Additionally, the melanocortin system is an important mediator of the leptin- and insulin-induced forms of hypertension. Of great clinical importance is also the role of MC3R activation in preventing ventricular tachycardia and fibrillation in myocardial ischemia, as well as MC4R agonism for the treatment and recovery from cerebral ischemia. Thus, the central melanocortin system appears as a pivotal pharmacological target for the treatment of a broad range of cardiovascular diseases, and a better understanding of how its components influence and regulate cardiovascular functions is of central importance for relieving the socioeconomic burden of such pathological conditions.

## Author Contributions

FC and JK wrote the manuscript. FC designed and prepared the figure. SD developed the concept and outline and edited the manuscript. All authors contributed to the manuscript and approved the submission.

## Conflict of Interest

The authors declare that the research was conducted in the absence of any commercial or financial relationships that could be construed as a potential conflict of interest.

## Publisher’s Note

All claims expressed in this article are solely those of the authors and do not necessarily represent those of their affiliated organizations, or those of the publisher, the editors and the reviewers. Any product that may be evaluated in this article, or claim that may be made by its manufacturer, is not guaranteed or endorsed by the publisher.
